# How Adolescents Use Technology for Health Information: Implications for Health Professionals from Focus Group Studies

**DOI:** 10.2196/jmir.5.4.e32

**Published:** 2003-12-18

**Authors:** Harvey Skinner, Sherry Biscope, Blake Poland, Eudice Goldberg

**Affiliations:** ^1^Department of Public Health SciencesUniversity of TorontoToronto ONCanada; ^2^Hospital for Sick ChildrenToronto ONCanada; ^3^Department of PaediatricsUniversity of TorontoToronto ONCanada

**Keywords:** Adolescent health, adolescent health services, health information, eHealth, information technology, Internet

## Abstract

**Background:**

Adolescents present many challenges in providing them effective preventive services and health care. Yet, they are typically the early adopters of new technology (eg, the Internet). This creates important opportunities for engaging youths via eHealth.

**Objective:**

To describe how adolescents use technology for their health-information needs, identify the challenges they face, and highlight some emerging roles of health professionals regarding eHealth services for adolescents.

**Methods:**

Using an inductive qualitative research design, 27 focus groups were conducted in Ontario, Canada. The 210 participants (55% female, 45% male; median age 16 years) were selected to reflect diversity in age, sex, geographic location, cultural identity, and risk. An 8-person team analyzed and coded the data according to major themes.

**Results:**

Study participants most-frequently sought or distributed information related to school (89%), interacting with friends (85%), social concerns (85%), specific medical conditions (67%), body image and nutrition (63%), violence and personal safety (59%), and sexual health (56%). Finding personally-relevant, high-quality information was a pivotal challenge that has ramifications on the depth and types of information that adolescents can find to answer their health questions. Privacy in accessing information technology was a second key challenge. Participants reported using technologies that clustered into 4 domains along a continuum from highly-interactive to fixed information sources: (1) personal communication: telephone, cell phone, and pager; (2) social communication: e-mail, instant messaging, chat, and bulletin boards; (3) interactive environments: Web sites, search engines, and computers; and (4) unidirectional sources: television, radio, and print. Three emerging roles for health professionals in eHealth include: (1) providing an interface for adolescents with technology and assisting them in finding pertinent information sources; (2) enhancing connection to youths by extending ways and times when practitioners are available; and (3) fostering critical appraisal skills among youths for evaluating the quality of health information.

**Conclusions:**

This study helps illuminate adolescent health-information needs, their use of information technologies, and emerging roles for health professionals. The findings can inform the design and more-effective use of eHealth applications for adolescent populations.

## Introduction

Health practitioners face several important challenges with adolescents. Adolescence is the developmental stage when health-risk behaviors may be initiated (eg, smoking, drug use, physical inactivity, high-risk sexual behavior, and not wearing protective gear), and when youths move from parental control to establishing their own separate relationships with health professionals [1]. However, youths can be difficult to engage in health care and health promotion, despite having access to more health information than in the past. Studies show that adolescents want to discuss issues with health professionals, but often they do not. For example, Klein and Wilson found in a national (United States) sample of adolescent boys and girls that the majority (70.9%) report at least 1 of 8 potential health risks, but most (63%) had not spoken to their doctor about any of these [2].

On the other hand, adolescents are typically the early adopters of new technologies. The Internet, in particular, provides innovative opportunities for engaging youths, including hard-to-reach populations (eg, youths in rural settings and street-involved youths) and those turned off by traditional health-education approaches. Youths' traditional sources of health information are no longer satisfying their needs, and they are increasingly using the Internet for health-related information [3,4]. A distinct advantage of the Internet is the potential for enhanced outreach in providing eHealth services to the community. Woodruff et al [5] provided initial data regarding the acceptability and impact of an Internet-based chat room for rural teen smokers. Skinner et al [6-8] developed a comprehensive eHealth Web site for youths based on the concept of a virtual island called CyberIsle, which includes an online teen clinic and behavior-change interventions such as smoking prevention and cessation [9].

As health-information sources on the Internet proliferate, concern is being expressed about the quality of this information [10,11] and about difficulties young people have in finding answers to their specific questions [12]. Ho and Lee [13] found a fairly-complicated relationship between computer use and youths' gender and lifestyle. Skinner et al [14] found that the quality of Internet access is not equal and that it greatly influenced young people's ability to obtain health information and resources. Internet-use statistics do not reflect this characteristic. In addition, filtering can restrict access for youths to health information. In a study of pornographic-material filtering, Richardson et al [15] found that at the least-restrictive level software filtered out 87% of erotic Net sites yet blocked 1.4% of health-information sites, and at more-restrictive levels the filtering blocked from 5% to 25% of health-related sites.

Research is illuminating issues about how searches are conducted for information on the Internet. In an observational study of 16 adult subjects, Eysenbach and Kohler [16] found that only 9 participants ever looked beyond the first search pages and 5 of them ever clicked a link on those pages. Hansen et al [17] studied how adolescents search for information using the Internet and found that they typically used a trial-and-error approach and did not consider the source of the content. Using simple search terms on popular search sites for information on smoking cessation for teens, Koo and Skinner [18] found that only 14 of the first 30 retrieved sites were of direct relevance to teen smoking cessation.

The aims of this study were threefold: (1) to identify particular needs that adolescents seek health information about, (2) to analyze how adolescents use various technologies for getting this information, and (3) to examine roles that youths see health professionals playing in linking technology and health information. Based on these findings, a framework is presented for integrating different technologies and information functions in eHealth applications for adolescents.

## Methods

Focus group methodology [19] was used to engage youths in discussions about their health-information and social-support needs, as well as the role that technology plays in addressing these needs. Our aim was to learn about how and why adolescents from diverse cultural, geographic, and socio-economic backgrounds access health information. The open-discussion format allowed youths to share episodes from their lives without prompting.

### Subjects and Site Selection

Twenty-seven focus groups were conducted with 210 youths from across Ontario, Canada; 55% were female and 45% were male. The median age of participants was 16 years (range, 10-28 years). Initial contacts were made with agencies serving youths (health agencies, community centers, drop-in centers, and schools), through a snowball sampling technique that involved obtaining subjects through chain referrals based on an extended network of relationships and contacts across the province. The majority of the focus groups were conducted with preexisting youth groups or in locations where youths congregated for programs. The median age difference within groups was 5.7 years (range, 0-11 years). The few older participants were from the street-involved and Aboriginal focus groups. Consistent with maximum variation sampling [20] in qualitative research, a sampling frame was developed to ensure diversity in terms of age, sex, geographic region of the province, and ethno-racial identity. Stratified sampling using a multistage sampling frame allowed for the inclusion of traditionally underrepresented youths, specifically street-involved youths, youths with physical disabilities, Aboriginal youths, first-generation Canadians, and newly-arrived Canadians. Slightly more than one third of the group sites (10) represented high-risk populations (eg, street-involved). The ethnic representation of the participants was: 28% North, Central, and South American; 22% European; 22% African and Caribbean; 14% pan-Asian; 7% Aboriginal; and 7% not stated. The focus group geographic-location settings were: 3 rural, 3 northern, 4 small urban, and 17 large urban.

### Focus Group Process

The focus groups were, on average, 90 minutes long. To provide consistency, the same TeenNet research associate (SB) who was not known by the participants facilitated all groups. Each focus group site provided a known cofacilitator to enhance participant's comfort, translate the study into terms uniquely understandable to each group, and to help draw out the youths to share their experiences. To reinforce the safety and confidentiality of focus group members, it was agreed that topics discussed in the group would remain confidential unless they impacted an individual's immediate safety. All participants were informed that if immediate safety was a concern the cofacilitator would follow up with the individual. Standard procedures were employed for obtaining informed consent (approved by University of Toronto's Human Subjects Review Committee). Parent or guardian consent was obtained in cases where participants under 18 years of age were not living independently and the focus group site was not a drop-in center.

A warm-up session had each group brainstorm about definitions of health. Initial work showed that unless a broad definition of health was grounded in participants' lives, many of the participants would respond with a narrow focus on health as being either the presence or absence of disease. In the focus groups, youths were asked to share experiences of using information technologies to address: (1) finding health information for self or others, (2) supporting personal change, (3) finding or providing social support, and (4) facilitating collective action. The focus group questions were derived in consultation with 3 committees: (1) selected youths, (2) frontline staff from youth agencies, and (3) a research advisory group. Two pilot groups were conducted to refine question wording and sequence prior to commencing the main study.

### Data Analyses

The audio of all focus-group interactions was tape recorded and transcribed. Several procedures were employed to maximize transcription quality, and to ensure that quality standards were maintained [21]. Verification of the accuracy of the transcriptions was achieved by randomly cross-checking the transcripts against the tapes [22]. Analysis followed a modified grounded-theory approach [23], where a selective coding template was developed based on major data themes [24]. The template was refined and extended following trial application to a cross section of transcripts. The coding template was peer reviewed [25] by the 3 committees and applied to all 27 transcripts using QSR N6 software [26]. Out of the approximately 60 nodes, this article focuses on the 12 technology-related nodes. The 12 nodes were reviewed by a group of 8 researchers for consistency and analyzed for categories, themes, and issues. In weekly analysis meetings, members discussed prepared notes on key themes, issues, and gaps related to a specific technology [27]. Categories, themes, and issues that were common to all the technologies were identified in the final phase of analysis. These were summarized into tables and figures with participant quotes used to illustrate the youths' voices. This analysis identified distinct trends in how youths were using different types of information technologies. As a final step, summary data were presented to a small sampling of cofacilitators for a modified member's check [28].

## Results

### Health-Information Needs

Table 1 summarizes the main issues raised by youths in this study in terms of expressed needs for health information. Table 1 lists the number of groups that raised a particular issue (Coverage) and the amount of time spent discussing a theme (Volume) measured by the number of coded single-line text units. Presenting the data in this format helps portray where health issues fit within the broader realm of adolescent life.

Regarding general health, study participants most-frequently used technology and traditional sources to find information about specific medical conditions and diseases (67%), followed by body image and nutrition (63%), violence and personal safety (59%), and sexual health (56%). The discussion was most extensive around the topic of violence and personal safety (1861 text units). In comparison to physical health, mental health issues were discussed much less often, with suicide and depression (22%) being the most-common examples. Study participants reported having health-information needs related to school (89%); interacting with friends (85%); and finding information about social concerns regarding income, housing, poverty, and employment (85%). Virtually all groups talked about action including personal change. Although study participants discussed mental-health issues less frequently, suicide and depression were an important theme for 22% of the groups.

### Concerns

Study participants raised some key issues about using the Internet to find health information. Quality was discussed as pivotal by all but 1 group (96% of the groups)—having ramifications on the depth and types of information that adolescents can find to answer their health questions. Finding personally-relevant health information was seen to be dependent on Internet-searching skills. Participants reported that they tend to use simple 1-word searches and did not dig deeply into search-engine results pages. Acquiring search skills was seen as dependent on Internet access, including the amount of Internet time available, quality of connectivity (bandwidth), and computer software. A common concern was the ability of the Web resource to answer their specific health related question.

### Linking Technologies With Functions

**Figure 1:**
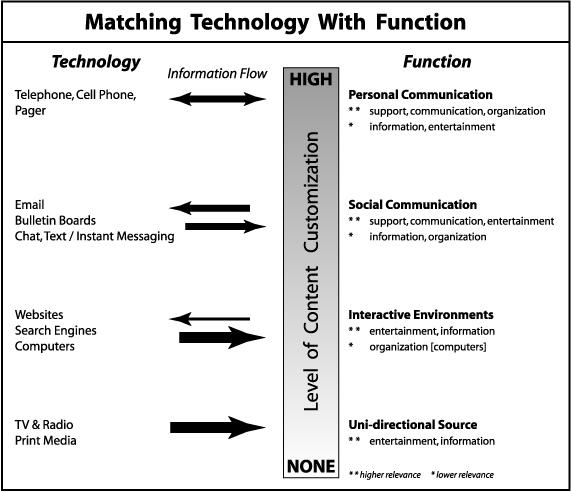
Matching technology with functions that meet the perceived health-information needs of adolescents

Study participants reported using various technologies for health information, ranging from traditional formats (television, radio, and printed material) to new venues such as mobile phones and interactive Web sites. Figure 1 provides a graphic synthesis that maps the relevance of the different technologies for meeting the perceived needs of adolescents. The technologies identified by study participants clustered into 4 domains that are differentiated in Figure 1 along a continuum ranging from highly interactive (high level of content customization) to fixed sources (no content customization). Table 2 gives a detailed description of how study participants use the 12 different technologies and the challenges they experience with them.

**Table 2:** Adolescents use of different types of technology

**Technology**	**Scope Of Use**	**Challenges**
1. Personal communication
Cell phone	Most often stated purpose was for personal safety Frequently used to make social plans once adolescents are out of the house	Big concerns about theft and loss of cell phones Concern about the privacy of cell phones Money and debt management Health impacts of using cell phones
Pager	Safety, privacy, and screening—can choose when they talk to the person Control of who you talk to—calls coming into adolescents' pagers are only for them	Fear of losing pager Pagers are identified with drug dealers and the poor
Telephone	Extensively used for social connection and gossip Increased credibility for Web sites that offer a contact phone number Contact professionals for information and appointments Use 1-800 (ie, toll-free) numbers	Extra cost for phone services to rural communities Issues with trust in accepting help lines: statement of confidentiality Help lines and information lines that use automated menus are frustrating
2. Social communication
E-mail	Keeping in touch with people they know Source of emotional support Can be easier to write out a personal problem than talk about it Petitions, subscribing to updates, and newsletters	Limited access to e-mail Concern over security of personal identity Fear of downloading viruses Unsolicited e-mail: advertising, junk, porn, and stalkers
Instant messaging (MSN Messenger and ICQ)	Keeping in touch with friends and people from school **Random chats with strangers** **On-going relationships with ICQ friends** Cybersex explorations	Don't know how to use or have access to ICQ Unsolicited porn and spam Cost of not having ICQ—being left out of group activities Fear of censorship and punishment
Bulletin board	Focused discussions, only respond to details shared Source of referrals and information for specific questions Mostly spoke with strangers	Like being anonymous and nonprejudicial Yet, some youths fear of having identity discovered
Chat room	Play and social interaction Linking with people with similar experiences and interests Recovery chat: support dealing with drug and alcohol problems	Access to chat software is limited Too much swearing Too many invitations for cybersex
3. Interactive environments
Web site	Internet is first stop for information Finding information about sensitive issues online Easy to find information on topics of personal interest eg, grooming, fashion, sports, and music	Avoiding the social costs of viewing pornography Access issues limit use at schools Difficult to find information for personal questions and school-related projects
Search engine	Only 5 or 6 search engines typically used **Tended to use 1-word searches** **Only looked at first page of results**	Either too many or not enough relevant sites identified Sites without relevant information turning up in the results page Old data turning up high in results page
Computer	Homework and the organization of information Computers allows adolescents to be more efficient and effective in school work	Typing is a barrier Family income affects quality of technology available to adolescents Information generated by adolescents is more appealing than adult-only created material
4. Unidirectional
Radio Television	Television is a source of credible general information MP3 use was common Keeping in touch with local news Source of music	Television is passive—can't control what you get Less current than Web sites
Print: Books Newspapers Magazines	Books are seen as one of the most-credible sources of information for serious projects and health projects Magazines are good sources of fun and adolescents' culture information (eg, body image, grooming, sports, and music)	Most paper media depends on literacy Takes time to find a book and they are usually in a library Relative speed compared to Web sites; can take too long to read for information

**Table 1:** Health-information needs raised by adolescents

**Theme**	**Coverage: Groups that Raised the Issue % (Number) N = 27**	**Volume: Number of Coded Single-Line Text Units**
General health		
Medical conditions	67% (18)	1252
Body image and nutrition	63% (17)	1168
Violence and personal safety	59% (16)	1861
Sexual health	56% (15)	1174
Drug use and drinking	44% (12)	752
Smoking	41% (11)	817
Mental health		
Suicide and depression	22% (6)	665
Stress	11% (3)	79
Grief and loss	7% (2)	42
Social		
School	89% (24)	4617
Income, housing, poverty, and employment	85% (23)	2604
Friends	85% (23)	1399
Music and gaming	78% (21)	882
Parents and family	74% (20)	626
Sports	63% (17)	1685
Action		
Collective action (volunteering, activism)	100% (27)	8782
Social support	96% (26)	9507
Personal change	96% (26)	7370
Health-information concerns		
Quality	96% (26)	4240
Trust	67% (18)	1819
Privacy	56% (15)	1104

#### Personal Communication

Cell phones were most often obtained for personal-safety reasons. Also, they were used to arrange social plans when the youths were away from the home phone. Virtually all types of personal conversations including health issues were considered appropriate when using a cell phone or telephone. The largest barrier with cell phones was cost of the unit, minute plans, and fear of debt; for example, ". . . so you're not building up a debt when you're only like sixteen years old." Mention of pagers was less common in the discussions, due to their negative association with the drug culture. A perceived strength common to pagers and cell phones was privacy. Telephones were almost invisible in the group discussions—some groups did not even consider them technology. The availability of toll-free numbers for health information was important to all youths. However, a major barrier to the use of toll-free support numbers was voiced by rural and northern youths, who were concerned that they were not eligible because of geographic isolation (a misperception). Geography also affected the availability of cell phone and pager service.

#### Social Communication

Study participants considered e-mail the most-accessible technology. It was used for everything from short messages and receiving health bulletins to providing and receiving emotional support. According to one youth: "like if you want to tell them something and you don't want to tell them over the phone or in person because it's kinda . . . hard to say." Finding and keeping an e-mail address was viewed by youths as very important. The largest barrier was related to timely and private access. Instant messaging (MSN Messenger and ICQ) was used mainly for social conversation with friends and "chat friends" formed in the virtual world—not as a venue for sharing feelings and personal disclosures about health-related concerns. However, access to instantaneous chat was not as common as e-mail. Bulletin boards were seen as a valuable source of health information because the anonymity provided an unbiased place to share personal information about health concerns; for example, ". . . read them all over and see which one is good." It also allowed participants to share expertise by providing advice without risking personal safety. Then again, some participants raised concerns about privacy and their ability to maintain anonymity in face of other's superior technological skill. Another concern was that information obtained from bulletin boards is of questionable quality. Chat rooms supported personal disclosure and participation in specialized topics ranging from sports and music to addictions; for example, "if you want advice, there's like advice chat room(s)." Group members were concerned about the amount of offensive content such as unsolicited sexual advances.

#### Interactive Environments

When using technology, Web sites were the first place that study participants looked for health information. However, important concerns about using Web sites were the consistency and quality of information. School-related information was perceived to be much more difficult to find on the Web than entertainment or social information. Unless a specific URL had been recommended to youths, they typically used their favorite search engine to find Web sites. However, search engines were often experienced as frustrating because of their tendency to uncover too few relevant or too many extraneous sites. One participant commented: "too many things to choose from. If you're looking for one site, at least twenty-five are gonna pop up that are completely different."

Participants found computers to be pivotal in their ability to perform work. Indeed, participants in our study strongly believed that not having a computer or possessing a slow system leaves youths with diminished ability to develop necessary computer skills and to perform in school. This point was underscored by the comment of one participant: ". . . if you don't have enough money to buy a computer you can't really use that stuff right?" Geographic issues impacted on using these technologies for health information. In particular, the limited availability of Internet service or broadband connectivity affected youths in relatively-small urban, rural, and northern communities.

#### Unidirectional Sources

Radio and television were mostly used for relaxation and entertainment because of the lack of control over content. According to one participant: "TV you can't ask a question." However, The Learning Channel was cited as a source of health information. Books were commonly used to verify information obtained from Web sites but only in cases involving a serious health issue or an important school assignment. Youths in this study tended to have greater trust about books as an information source; for example, ". . . they won't publish a book that has inaccurate information." Magazines were seen as easily-obtained credible sources of information. Magazines were described like Web sites: short, graphical, easy to digest, and immediately relevant. The largest barrier to using books and newspapers was literacy level.

#### Technology Functions

According to the thematic analyses, adolescents use the various technologies to serve 5 major functions (see Figure 1):

1. Entertainment: finding information about personal interests (eg, movies and sports), having fun in chat rooms, and playing virtual games
2. Information: gathering and sharing information for personal use and school work
3. Communication: interacting with friends and strangers
4. Organization: collaborating on projects and organizing people/events
5. Support: connecting with others to give or receive self support and mutual support.

Entertainment was the most frequent reason study participants used technologies. In addition, they used information technologies to answer health questions, become better informed, and share the resulting information with others. The availability of safe, appropriate opportunities to connect with others and create virtual support networks was highly valued. This connection was seen to provide a nonthreatening environment for discussing sensitive personal health concerns (eg, sexual activities). Although getting help and support with personal issues was mentioned least often by participants, strong opinions were voiced about the appropriateness of using social communication technologies in this way (eg, concerns about maintaining anonymity).

### Technology and Emerging Roles for Health Professionals

A higher-order analysis of the data focused on studying youths' perspectives about technology and the role of health professionals in their lives. Three emerging roles were identified (Table 3).

First, practitioners and health care settings can provide a major interface with eHealth technology and applications. Youths looked to practitioners for assistance in finding and evaluating information about a particular health need. For example, "go to your doctors and ask them if they . . . can point you where to go."

Second, eHealth technology can enhance interactions and personal connection of adolescents with health practitioners. One study participant described a situation where "instead of going to the doctor I went on the Internet . . . afterwards I went to the doctor because I didn't think the Internet helped me that much." Technology can extend the ways and times when practitioners are available—enabling them to be more approachable for adolescents around their health concerns.

Third, health practitioners can play a major role helping youths build critical appraisal skills for evaluating the quality of health information found through eHealth sources. This need was stated by one youth: "go to the Internet for quick information . . . but knowing that it shouldn't be trusted."

**Table 3:** Emerging roles for practitioners and health care settings in eHealth

1. Providing a technology interface and direction: Provide a key interface for adolescents with eHealth resources Assist and augment adolescents in their access to quality health information Serve as an important backup resource to eHealth information Provide direction on where to get further information and assistance
2. Enhancing connection and trust: eHealth provides practitioners with an entry to build relationships and trust with their young patients eHealth enables practitioners to be more engaging with adolescents and increase their readiness to look at personal health issues Technology can extend times when and venues where practitioners are available
3. Fostering critical appraisal: Help adolescents develop skills for assessing the quality of health information Encourage critical perspectives about health information and eHealth sources Encourage and help adolescents develop digital-literacy skills

## Discussion

Searching for health information using eHealth technology can seem to adolescent health consumers like running in a maze. A key concern identified in this study was being overwhelmed by information, yet not being able to get a specific question answered. This frustration was expressed quite succinctly by one youth: "it can get just overwhelming on just the number of sites that have nothing really to do with what you're looking for." Adolescents frequently make health-related decisions in isolation from traditional health sources. However, study participants reported that they find this task difficult and want better support.

Internet technologies could be used to augment gaps when traditional venues for health information are less available (eg, professionals) or perceived to be less helpful (eg, pamphlets). Many adolescents prefer using information technology to traditional sources in situations that may cause embarrassment with peers or conflict with parents or teachers. Bulletin boards and specialized chat rooms are popular places to pose questions and gather information. Adolescents indicated that their peers (online and off) are primary sources of health information. However, this raises concern because of the "personal" nature of information shared. Adolescents indicated they turn to Internet-based health resources because of its 24-hours-per-day availability, and its lack of perceived judgment and conflict. Yet, there was considerable debate among study participants about the appropriateness of this venue for sharing personal health information.

Study youths indicated that they would be open to increased interaction and support from health practitioners. They saw practitioners as reliable experts on health information, but noted barriers to having timely access to them. They were aware that health practitioners have an expertise in both assessing and finding quality health information. This was seen as very important because participants acknowledged gaps in their skills (eg, sorting through "too many" information sources from a search engine request), especially when looking for specialized and personal health information. They were receptive to health professionals using their expertise to help them bridge the gap between information they are currently finding and the potentially higher-quality health information available on the Internet. For example, practitioners could help by: recommending Web sites for specific health issues, giving advice about topic search strategies, and providing guidance on critical appraisal of information found.

Whereas one of the biggest draws of the Internet is that it is potentially available at all times (24 hours per day, 7 days per week), a major limitation described by study participants was access to health professionals—eg, they are only available by appointment. For adolescents living in small communities another barrier was privacy. According to one participant: "You can't even trust a doctor or anyone in a small town - they are professional but they also live here." The nature of small towns raises concerns about health professionals inadvertently linking requests for sensitive information back to adolescents' social networks (especially parents). This vulnerability left some youths hesitant to approach practitioners about potentially-embarrassing topics.

New and expanding roles are emerging for health professionals to integrate eHealth resources into their clinical practice and community outreach. The map (Figure 1) summarizes how study participants used the various information technologies in performing 5 common functions. It underscores a key point for practitioners and developers of eHealth applications—"health" is not a primary concern of most youths. One needs to go where they are (entertainment, communication, and organization functions) as a stepping-stone to health issues (information and support functions). The map can help practitioners understand how their young patients use technologies in their daily lives. Also, the map can help guide eHealth program development in matching appropriate technologies to health-information needs of adolescent populations.

In conclusion, this study underscores the many challenges adolescents face in getting quality health information using technology. The findings provide a better understanding about the health-information needs and concerns of youths, and the ways that they use various technologies. At the same time, the study helps illuminate some enhanced and innovative roles for practitioners and health care settings in better serving the needs of adolescents via eHealth.
